# Cyclosporin A Associated Helicase-Like Protein Facilitates the Association of Hepatitis C Virus RNA Polymerase with Its Cellular Cyclophilin B

**DOI:** 10.1371/journal.pone.0018285

**Published:** 2011-04-29

**Authors:** Kengo Morohashi, Hiroeki Sahara, Koichi Watashi, Kazuki Iwabata, Takashi Sunoki, Kouji Kuramochi, Kaori Takakusagi, Hiroki Miyashita, Noriyuki Sato, Atsushi Tanabe, Kunitada Shimotohno, Susumu Kobayashi, Kengo Sakaguchi, Fumio Sugawara

**Affiliations:** 1 Genome and Drug Research Center, Tokyo University of Science, Noda, Chiba, Japan; 2 Laboratory of Biology, Azabu University School of Veterinary Medicine, Sagamihara, Kanagawa, Japan; 3 Department of Virology II, National Institute of Infectious Diseases, Shinjuku-ku, Tokyo, Japan; 4 Department of Pathology, Sapporo Medical University School of Medicine, Sapporo, Hokkaido, Japan; 5 Research Institute, Chiba Institute of Technology, Narashino, Chiba, Japan; Washington University, United States of America

## Abstract

**Background:**

Cyclosporin A (CsA) is well known as an immunosuppressive drug useful for allogeneic transplantation. It has been reported that CsA inhibits hepatitis C virus (HCV) genome replication, which indicates that cellular targets of CsA regulate the viral replication. However, the regulation mechanisms of HCV replication governed by CsA target proteins have not been fully understood.

**Principal Findings:**

Here we show a chemical biology approach that elucidates a novel mechanism of HCV replication. We developed a phage display screening to investigate compound-peptide interaction and identified a novel cellular target molecule of CsA. This protein, named CsA associated helicase-like protein (CAHL), possessed RNA-dependent ATPase activity that was negated by treatment with CsA. The downregulation of CAHL in the cells resulted in a decrease of HCV genome replication. CAHL formed a complex with HCV-derived RNA polymerase NS5B and host-derived cyclophilin B (CyPB), known as a cellular cofactor for HCV replication, to regulate NS5B-CyPB interaction.

**Conclusions:**

We found a cellular factor, CAHL, as CsA associated helicase-like protein, which would form trimer complex with CyPB and NS5B of HCV. The strategy using a chemical compound and identifying its target molecule by our phage display analysis is useful to reveal a novel mechanism underlying cellular and viral physiology.

## Introduction

Cyclosporin A (CsA) possesses immunosuppressive effects and is widely used for allogeneic transplantation [1]. These therapeutic effects of CsA, in particular downregulation of interleukin 2 (IL-2) production by T cells, are considered to be responsible for the suppression of immunological events via cellular immunology [2], [3]. Its mechanism is widely believed to include CsA binding to its primary cytoplasmic receptor cyclophilin A (CyPA). This CsA/CyPA complex inhibits the phosphatase activity of calcineurin, which is essential for the activation of nuclear factor of activated T cells (NFAT) transcription factors and their downstream cytokine production [2]–[5]. The cyclophilins (CyP), identified as cytoplasmic receptors for CsA are a family of peptidylprolyl cis-trans isomerases (PPlase) and include more than ten subtypes [6]–[8]. Recently, it was reported that several CyPs regulated hepatitis C virus (HCV) replication; CyPA binds to HCV NS5A and NS5B proteins. CyPB also interacted with HCV NS5A and NS5B [9]–[11]. The interaction of CyPB stimulates the RNA binding activity of NS5B. These viral-cellular interaction mechanisms were revealed by a chemical biological analysis focusing on an anti-viral characteristic of CsA. However, it has not been fully understood how a series of CsA-target proteins regulate HCV replication. We obtained the data suggesting the possibility that CsA target factor(s) other than CyP family also modify HCV replication.

To exploit a novel drug target is a challenging but a powerful strategy to elucidate unknown aspects of cellular physiology that are modified by the compound. In this study, we identify a CsA binding factor by a phage display method. There are various methods to isolate targets of small molecules. Most of the methods, however, require tagged small molecules for screening to separate the drug and protein complex. The steps to synthesize tagged small molecules are technically limited in the case of complicated molecules such as CsA. To overcome this limitation, we recently developed a labeling method that can be theoretically utilized for any chemical substance [12]. A highly reactive carbene induced by UV irradiation reacts with CsA, resulting in the production of immobilized CsA in a nonspecific manner. By using the photoaffinity method, we successfully immobilized CsA on resins and performed phage display screening. This method cloned a CsA associated helicase-like protein, which we termed CAHL, and this protein was shown to interact with HCV replication machinery. Our result presents an example for the chemical biological method that could facilitate to reveal a mechanism of viral-cellular interaction.

## Results

### Phage display screening with immobilized CsA isolated CsA associated helicase-like protein, CAHL

**Figure 1:**
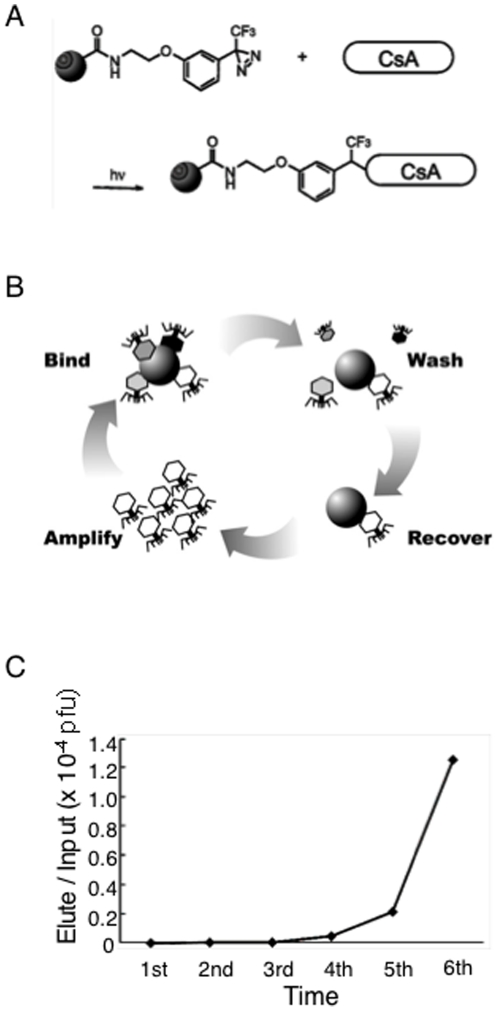
Immobilization of CsA and phage display screening. (A) A schematic diagram of CsA immobilization on photoaffinity resins. (B) Procedure of phage display screening. (C) Relative enrichment of phage particles. Relative enrichment was determined by the relationship between phage titer of elution from a CsA immobilized resins and input.

To explore CsA binding proteins, we applied a chemical biology approach. In general, small molecule is necessary to be chemically modified such as biotinylated to be immobilized on solid surface for isolation of binding proteins. However, due to the structural complexity of CsA, it is technically challenging to chemically modify a certain residue of CsA. Therefore, we took advantage of photoaffinity coupling method, which we previously developed [12]. The highly reactive carbene induced by UV irradiation reacted with CsA, resulting in the production of immobilized CsA on solid surface in a nonspecific manner (Fig. 1A). We performed phage display screening with multiple cycles that consist of binding, washing, recovery and amplification (Fig. 1B). We used phage particles randomly displayed 12 amino acids as a library [13]. Through the screening cycles, the ratios of eluted phage particles associated with CsA-immobilized resins comparing to input were dramatically increased (Fig. 1C). We randomly picked up 22 single phage clones from the sixth panning elution (Table S1). Five out of the 22 phage clones were identical, and we called it phage #13. In order to validate the binding specificity of the phage, we amplified phage #13 and measure the ratio of eluted phage titer with CsA and mock resins, which were treated with MeOH to block photoaffinity reaction. The ratio of the phage #13 was 3.75, whereas randomly picked up phage was 1.00. These results indicated that the phage #13 specifically associated with CsA-immobilized resins.

### CAHL has an RNA-dependent ATP hydrolysis activity

**Figure 2:**
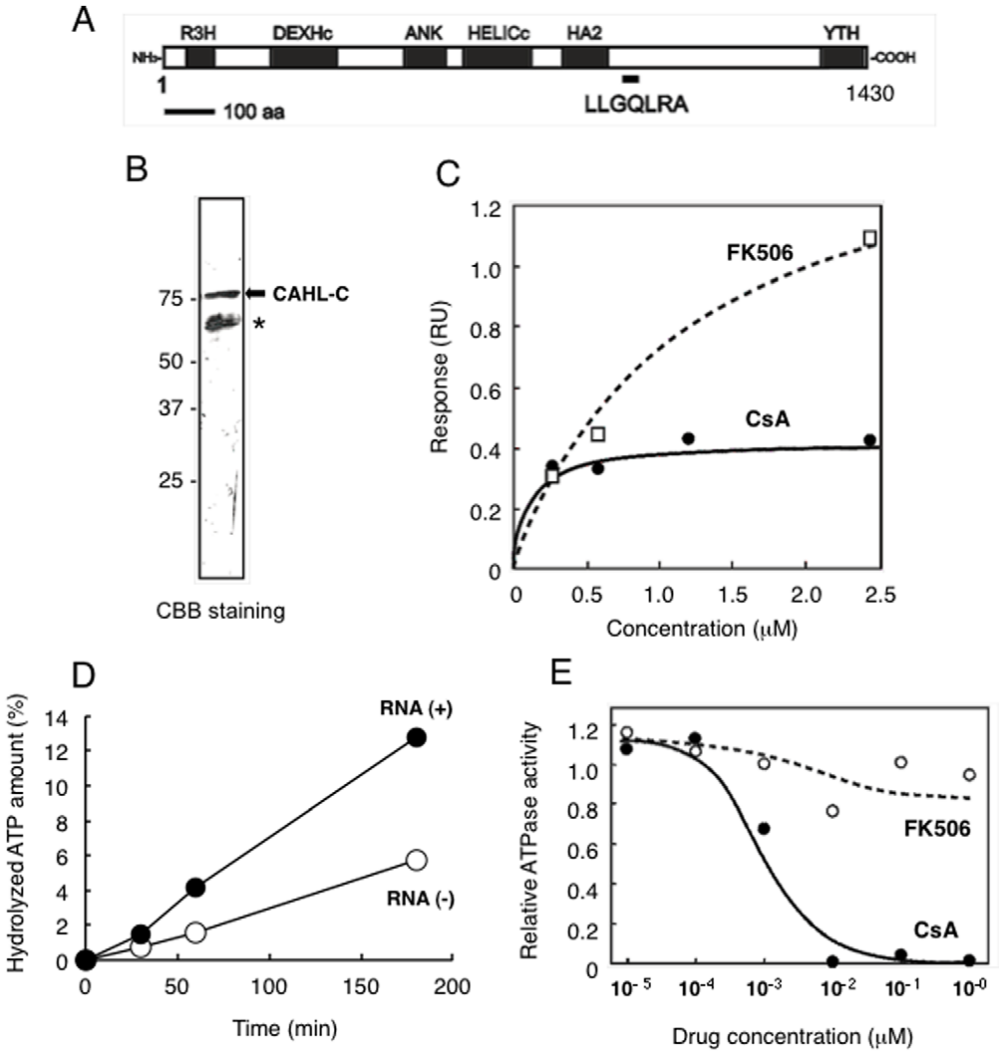
Cloning of CsA associated helicase-like protein (CAHL) by phage display screening. (A) Schematic representation of CAHL protein. R3H (*cd*02325), DEXHc (*cd*00269), ANK (*cd*00204), HELICc (*smart*00490), HA2 (*pfam*04408) and YTH (*pfam*04146) motifs were found by a CD search (http://www.ncbi.nlm.nih.gov/Structure/edd/wrpsb.cgi). Underline indicates a region of the LLGQRA amino acid sequence identical to the CsA-associated sequence displayed on phage #13. (B) Purified recombinant CAHL-C protein was confirmed by SDS-PAGE analysis (arrow). Asterisk indicates degraded products. (C) A kinetic plot and binding isotherm for binding of CsA (closed circle) and FK506 (opened square) to CAHL-C sensor chips in concentrations ranging from 0.25 to 2.5 mM. The estimated KD value of interaction between CAHL-C and CsA or FK506 was 1.2×10^−7^ or 2.5×10^−6^ M, respectively. (D) RNA-dependent ATP hydrolytic activities of CAHL. Filled and open circles indicate ATP hydrolytic activities of CAHL in the presence (closed circle) or absence (opened circle) of total RNA extracted from liver cells, respectively. (E) CsA inhibitory effects on ATP hydrolytic activities of CAHL. Filled and open circles indicate ATP hydrolytic activities with CsA (closed circle) or FK506 (opened circle), respectively.

Phage #13 was predicted to display amino acids, LVFGTLLGQLRA, in the carboxyl terminus of its phage-coat protein, which is responsible for interaction with CsA. We searched the protein database to find proteins that showed similarities to the LVFGTLLGQLRA sequence. As a result of the search, we found a protein with a sequence identical to LLGQLRA, encoded by a gene accession number NM_022828 in the NCBI database. NM_022828 is predicted to encodes 1430 amino acid protein that has a couple of conserved domains, such as DEXHc helicase, RNA-dependent ATPase and ankyrin repeat (Fig. 2A; Fig S1). LLGQLRA sequence is located in the middle region of the protein (amino acids 940–946), where is no known conserved motif is found (Fig. 2A). Since it has not been reported on its biological functions, hereafter we refer to the NM_022828 as CsA-associated helicase-like protein, CAHL. To confirm the interaction between CAHL and CsA, we prepared a recombinant C-terminal protein of CAHL (named CAHL-C) that consisted of amino acids 761 to 1430 (Fig. 2B) including LLGQLRA motif, and performed surface plasmon resonance (SPR) analysis. It was difficult to use a full-length CAHL for *in vitro* pull-down assays since obtaining enough amount of full-length CAHL for SPR was technically challenging due to high insolubility. Considering that CsA binding sequence found by the phage display screening is located C-terminus of CAHL, we used CAHL-C protein. A specific binding response with CsA was observed (KD = 1.2×10^-7^ M), whereas those with FK506, which is an immunosuppressant and has no HCV-inhibitory activity, were significantly weak (KD = 2.5×10^-6^ M) (Fig. 2C). Since CAHL was predicted to be RNA-dependent ATPase based on conserved domains (Fig. 2A), we measured the ATPase activity of CAHL in the presence and absence of RNA. As shown in Fig.2D, RNA-dependent ATP hydrolytic activity of CAHL-C was clearly observed, and this activity was suppressed in a dose-dependent manner when CsA, but not FK506 (Fig. 2E). A difference of inhibitory effects of CsA and FK506 on CAHL-C hydrolytic activity is more than 2-orders of magnitude. Considering that the difference of *KD*s of CsA and FK506 values measured by SPR, CsA association with CAHL would significantly affect on the activity. These results indicate that CAHL had RNA-dependent ATPase activity and was specifically inhibited by CsA.

### CAHL is localized in ER and its expression is up-regulated by TNF-α treatment

**Figure 3:**
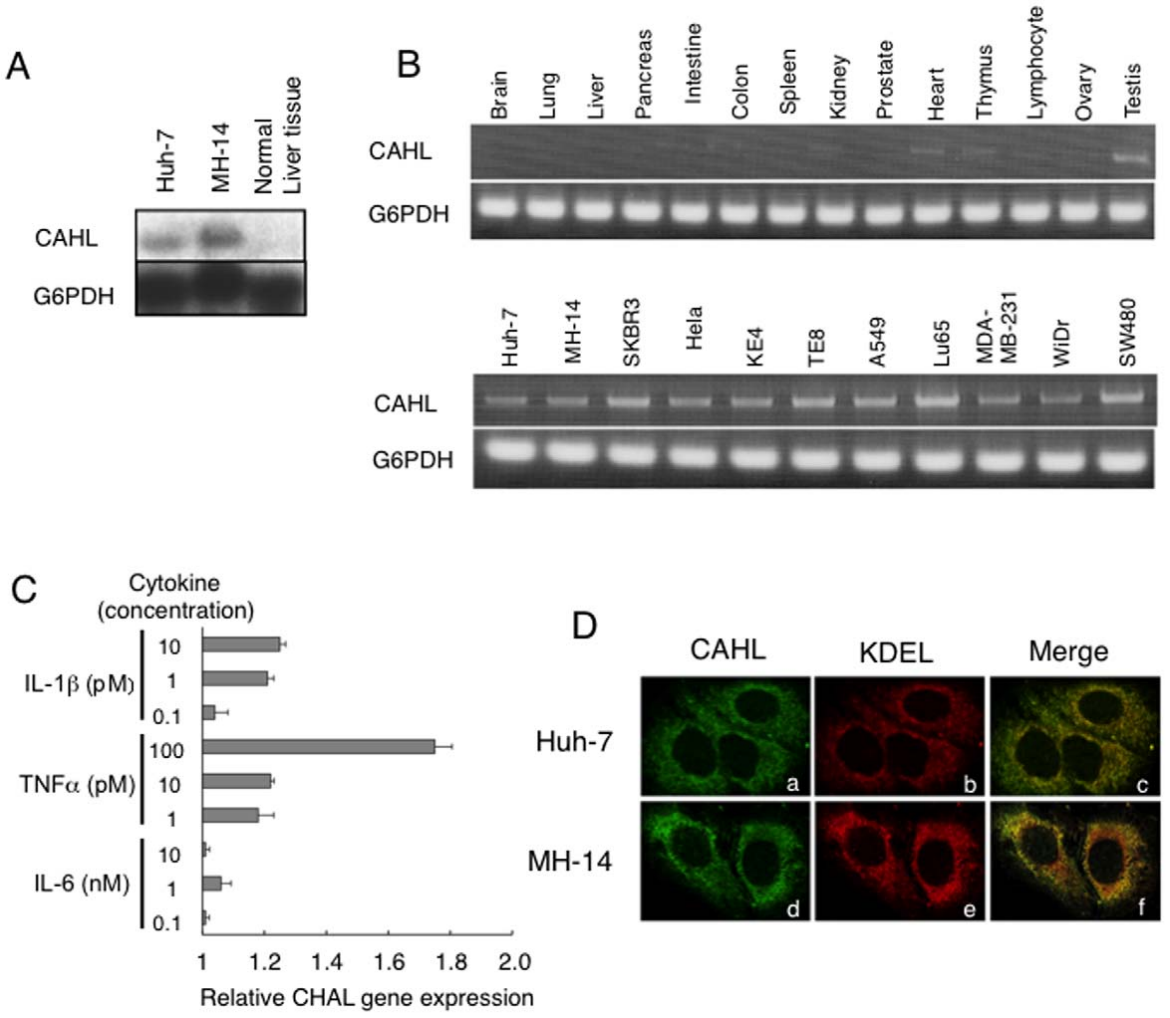
Expression profile of CAHL. (A) For Northern blot analysis, CAHL and G6PDH as an internal control were detected in RNAs derived from Huh-7 and MH-14 cells, as well as normal liver tissues. (B) RT-PCR analysis for CAHL, and G6PDH as an internal control, were performed using RNAs derived from normal human tissues and tumor cells. (C) CAHL in normal liver cells was upregulated by TNF-α. Normal liver cells were cultured in the presence of proinflammatory cytokines, IL-1β, TNF-α, and IL-6 at the indicated concentrations for 8 h. Subsequently, cells were harvested, and measurement of these cells derived-CAHL gene expression by quantitative analysis by was performed using the LightCycler system. These data represent as relative rates (1 =  non-treated cells). Error bars represent the standard error of the mean. (D) Colocalization of CAHL with KDEL as an endoplasmic reticulum (ER) marker. Indirect immunofluorescence analysis was performed on Huh-7 and MH-14 cells. Cells stained with anti-CAHL (panels a and d, green) and an anti-KDEL mAb (panels b and e, red) for ER identification as a marker used as a primary antibody followed by Alexa Fluor 488-conjugated goat anti-rabbit and 594-conjugated goat anti-mouse antibodies, respectively. Merged images of green and red signals are shown in panels c and f.The optically merged image is representative of most cells examined by laser confocal microscopy. Original magnification: x1000.

Since biological functions of CAHL were unknown, we investigated the biological background of the CAHL gene. We first performed RNA blotting analysis and RT-PCR using normal human tissues and tumor cells. As shown in Fig. 3A, CAHL-transcripts with approximately 1.6 kbp were detected in both human hepatoma Huh-7 cells and MH-14 cells, which do not and do carry the HCV subgenome replicon, respectively, whereas much less was detected in normal liver tissues. RT-PCR analysis revealed that in other normal tissues (though not testis) little or no expression of CAHL was observed as compared the house-keeping gene G6PDH, whereas clear expression of it was detected in all the tumor cell lines examined (Fig. 3B). Since the CAHL expression was very little in the normal liver tissues, a question was arisen: how is CAHL expression regulated? It is possible that CAHL could be induced by inflammation caused by virus infections. To test the hypothesis, we observed whether CAHL expressions are induced by inflammatory signals. Indeed, CAHL in normal liver cells was upregulated in the presence of a proinflammatory cytokine, tumor necrosis factor (TNF)-*α* with dose dependent manner (Fig. 3C), suggesting that CAHL can express to some extent in the liver under chronic hepatitis. Next, we observed CAHL subcellular localization in Huh-7 and MH-14 cells using an anti-CAHL antibody (Fig. 3D). Fluorescence derived from CAHL demonstrated that CAHL was co-localized with KDEL protein as a marker for endoplasmic reticulum (ER) in both the presence (MH-14 cells) and absence (Huh-7 cells) of the HCV subgenome, indicating that CAHL could localize in ER with HCV independent manner. Moreover, it was observed that CAHL also colocalized with HCV-derived proteins such as NS3, NS4A, NS4B, NS5A and NS5B localized in ER (Fig. S2). Thus, these data strongly suggested that CAHL would localize in ER.

### Association of CAHL, NS5B and CyPB

**Figure 4:**
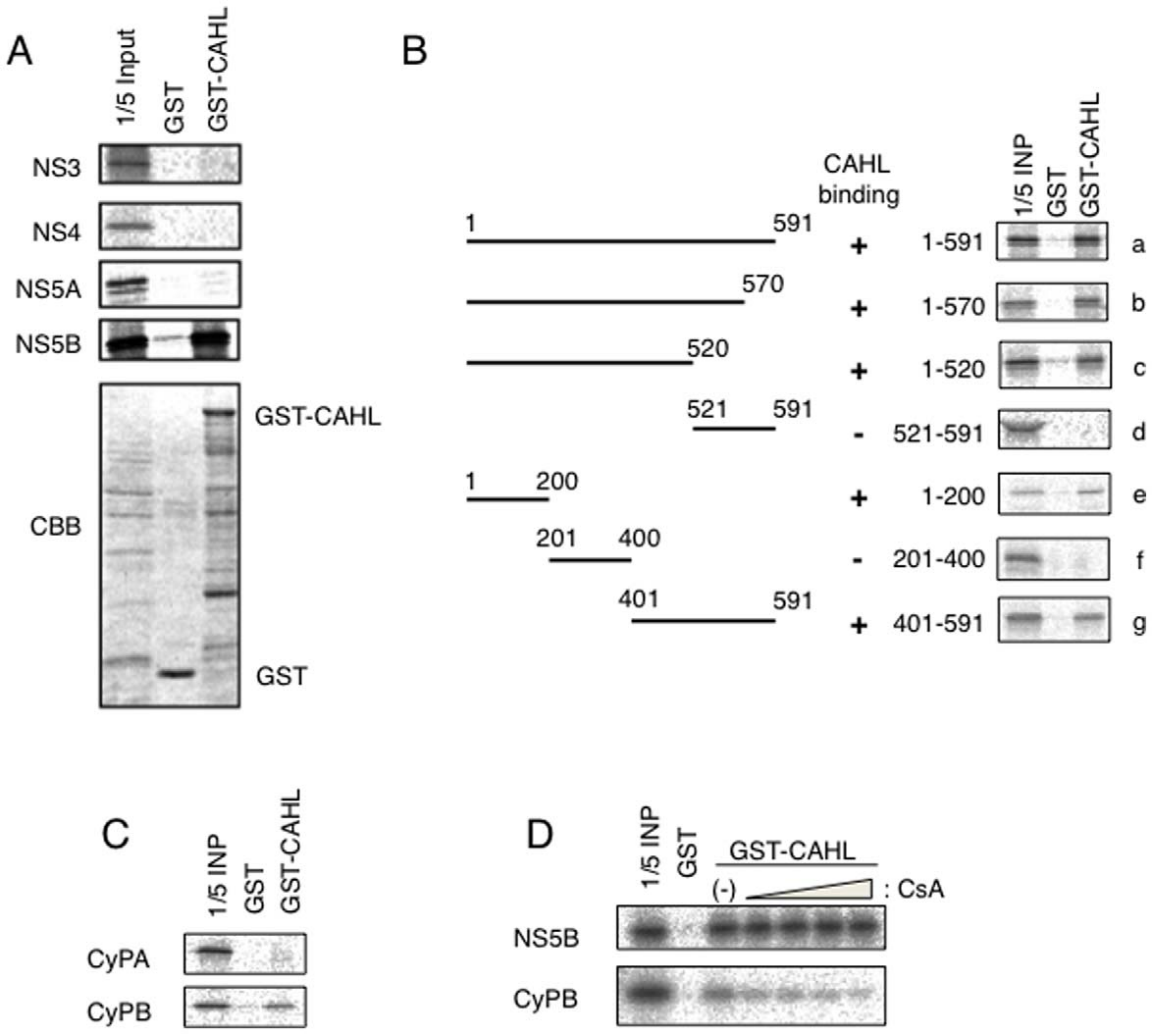
CAHL interacted with HCV NS5B and CyPB. (A) [^35^S]-labeled in vitro translation products of HCV NS3, NS4B, NS5A, and NS5B were incubated with a recombinant GST fusion protein of CAHL (GST-CAHL) or GST as a negative control. “1/5 input” designates the signal for 1/5 the amount of the [^35^S]-labeled product used in the pull-down assay. CBB staining patterns for the pulled-down proteins are shown in the bottom panel. (B) Mapping of the regions of NS5B responsible for the interaction with CAHL. At the left of the panel, schematic representations of the full-length and truncated mutants of NS5B are shown. The numbers indicate the amino acid residue numbers in NS5B. “CAHL binding” summarizes the results of the GST pull-down assay by +/−. GST pull-down data are presented as described in (A). (C) GST pull-down assay between GST-CAHL and in vitro translated CyPA or CyPB was performed as described in (A). (D) The interaction of CAHL with CyPB was disrupted by CsA treatment. GST pull-down assay between GST-CAHL and NS5B was performed in the absence and presence of CsA. The concentrations of CsA in lanes 4–7 are 1, 2, 8, and 20 µg/ml, respectively.

Since CAHL interacts CsA, which has inhibitory effects to HCV replication, it is interesting to investigate the molecular interactions of CAHL and HCV-derived molecules involving the replication machinery. Intriguingly, the purified full-length CAHL fused to GST was coprecipitated with NS5B but not NS3, NS4B or NS5A protein, as shown in Fig. 4A. To determine a regions of NS5B responsible for binding with CAHL, various dissected NS5B proteins were subjected to pull-down assays, resulting in that two separated regions (1–200 aa or 401–520 aa) of NS5B were sufficient for the interaction with CAHL (Fig. 4B). In addition to the CAHL and NS5B interaction, we found interaction between CAHL and CyPB, but not CyPA (Fig. 4C). The interaction of CAHL and CyPB was disrupted with presence of CsA, whereas the association of CAHL with NS5B was not (Fig. 4D). These results suggest that trimer complex consisting of CAHL, CyPB and NS5B could form.

### CAHL has a main role in HCV-replication via NS5B

**Figure 5:**
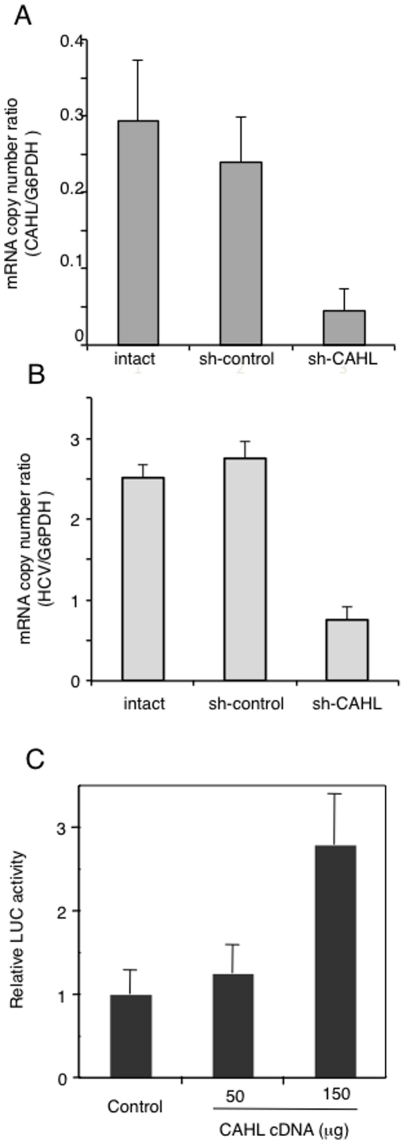
Establishment stably CAHL knockdown cell. (A) Lentivirus packed with shRNA against CAHL (sh-CAHL) or non-targeting shRNA (sh-control) were introduced into MH14 cells. Total RNAs were harvested and the absolute mRNA copy numbers of CAHL were examined by quantitative real time RT-PCR. (B) The same samples of total RNAs were used for the measurement of the absolute RNA copy number of the HCV genome by quantitative real time RT-PCR. (C) Cured MH-14 cells were transfected with LMH14 RNA reporter together with the expression plasmid for CAHL or the corresponding empty vector. At four days post-transfection, luciferase activities were measured. These results (A–C) represent the means of three independent experiments.

The finding that CAHL structurally associated with the CyPB/NS5B complex in cell-free assessment prompted us to examine whether this trimer complex could act for HCV genome replication *in vivo*. First, five small interference RNAs (siRNA) specific for the CAHL gene (si-1, -2, -3, -4, and -5) were individually transfected into MH-14 cells to examine RNA sequence induced effectively down-regulation. When si-3 siRNA was transfected into cells, the endogenous CAHL gene expression reduced approximately 90% compared with si-control (treatment with siRNA for non-target gene) (Fig. S3), and among them, si-3 induced down-regulation of CAHL gene expression most effectively. Subsequently, we applied short hairpin RNA (shRNA) technology to stably knockdown CAHL gene expression in MH14 cells. We cloned DNA oligo coding the effective siRNA against CAHL gene into pLKO.1-puro shRNA vector. Lentivirus packed with shRNA against CAHL (sh-CAHL) or non-targeting shRNA (sh-control) were introduced into MH14 cells, and then these cells were cultured in the presence puromycin. As a result, we successfully obtained stably CAHL gene knockdown cell line, which reduced approximately to 6-fold compared with sh-control (Fig. 5A). In these sh-CAHL cells, HCV RNA was decreased approximately to 4-fold less than that in the sh-control cells (Fig. 5B). Furthermore, ectopic expression of CAHL increased the HCV replication level in a dose-dependent manner (Fig. 5C). These results suggest that CAHL positively plays in HCV replication.

**Figure 6:**
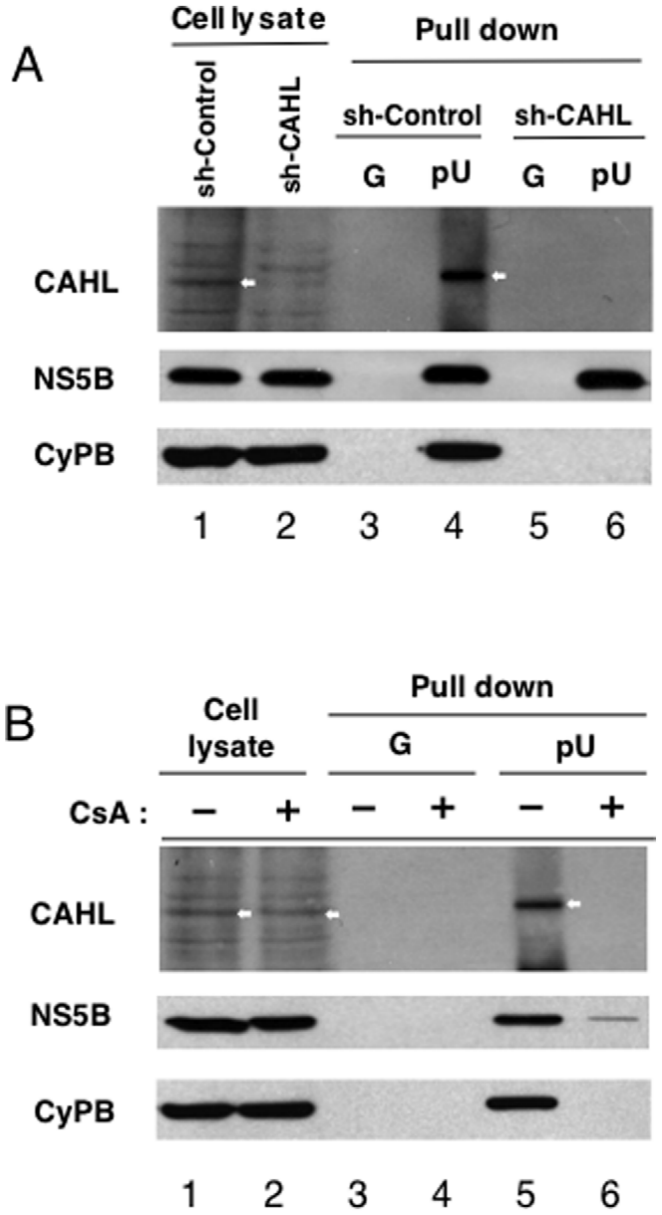
CAHL associated with the CyPB/NS5B complex plays critical roles in HCV replication. (A) Cells (sh-control or sh-CAHL cells) were harvested and analyzed protein expressions by using anti-CAHL, anti-NS5B, and anti-CyPB antibodies. White arrows indicate CAHL protein. (B) Cells were treated with or without 2 µg/ml CsA for 24 h, and then harvested and analyzed. These results were reproduced in three independent experiments. White arrows indicate CAHL protein.

To investigate the outcome of the interaction of CAHL with NS5B/CyPB, we performed RNA binding activity assay using sh-CAHL cells. NS5B is a viral RNA-dependent RNA polymerase and possesses RNA binding activity [11]. Indeed, NS5B formed a complex on RNA-immobilized sepharose together with CyPB and CAHL (lane 4 in Fig. 6A). However, shRNA-mediated depletion of endogenous CAHL dissociated CyPB from the NS5B/RNA complex (lane 6 in Fig. 6A), indicating that CAHL mediates the association of CyPB with NS5B/RNA. Moreover, when CsA was added to sh-CAHL cells, both CAHL and CyPB were dissociated from RNA (lane 6 in Fig. 6B). Thus, the possibility is suggested that the promotion of CyPB-NS5B complex association by CAHL is related with the stimulatory role of CAHL in HCV replication.

## Discussion

Phage display, invented by Smith and Petrenko, is a versatile method for the detection of small molecule-binding proteins [14]. The technique can also be used to identify binding sites within the target protein itself. The combination of screening a library of phage-displayed peptides and analysis of affinity-selected peptides is anticipated to become a powerful tool for identifying drug-binding sites [15]–[19]. Screening phage display libraries generally entails immobilizing the drug onto a solid surface [20]. In the conventional method of phage display, small molecules should be converted into biotinylated derivatives and immobilized on a streptavidin-coated matrix. Conventional immobilization requires the presence of desirable functional groups within the drug molecule as well as a multistep process to prepare the biotinylated derivatives. In contrast to biotinylation, photoimmobilization makes it possible to covalently immobilize drugs on a solid surface without the need for derivatization. We and Kanoh *et al*. have reported the affinity purification of proteins using affinity matrices, in which small molecules are photoimmobilized by photoreaction [12], [21]. Because the photoreaction proceeds in a functional group-independent manner, the molecules are immobilized onto the solid surface in a nonoriented fashion. Thus, photoimmobilization can be a useful tool for the comprehensive analysis of drug-binding proteins.

Using this method, we identified CAHL as a novel target protein for CsA. CsA is a natural compound showing multiple biological activities, including an immunosuppressive function, anti-chaperone activity, inhibition of transporter activity and anti-viral activity against human immunodeficiency virus and HCV. Thus far, p-glycoprotein and formyl peptide receptor, as well as CyPs, were reported as binding proteins for CsA [22], which enabled elucidation of the mechanism of the CsA-induced immunosuppressive function, anti-chaperone activity and anti-transporter activity, respectively. Although CyPA promotes HCV replication [11], [23], [24], we cannot fully explain the whole mode of action of CsA against HCV. CyPB is also reported to regulate HCV replication. It was reported that the HCV replicon showing resistance against the CsA-mediated anti-HCV effect possessed mutations in the coding region for NS5A and NS5B [25], [26], indicating that NS5B was one of the determinants for the sensitivity to CsA. However, some such mutations within the NS5B coding region were dropped outside the region interacting with CyPA and CyPB [11], [24], leading to the possibility that another cellular protein which is targeted by CsA, binds to NS5B and regulates HCV replication. The CAHL-NS5B regulation machinery is consistent with this idea. Deletion analysis for NS5B demonstrated that two separate regions (1-200aa and 401-520aa) of NS5B are likely to be involved in the interaction with CAHL. These regions are different from the NS5B domain interacting with CyPB (521-591aa) [11], suggesting that NS5B would interact with both CyPB and CAHL at the same time. Indeed, the mutations that induced resistant to CsA, the I432V in NS5B reside inside the regions interacting with CAHL (1-200 aa and 401-520 aa) [26], supported the relevance of CAHL in HCV genome replication. As another aspect, it is interesting that two CsA target molecules interact with each other and NS5B. Although we do not know in detail the implication of the interaction of these two CsA target molecules, CyPB and CAHL, there is a similar example already known: two FK506-binding proteins, P-glycoprotein (P-gp) and FKBP42, associate with each other [27]. In this situation, FKBP42 modulates P-gp function. We do not know in detail how these two target molecules of CsA, CyPB and CAHL both regulate NS5B function, which is a future subject of the study. Currently, a CsA derivative shows remarkable anti-HCV effect in chronic HCV-infected patients in the phase II clinical trial, and its mode of action needs to be fully clarified [28]. Our data suggest a new link of CAHL, in addition to CyP family, with CsA derivative's anti-HCV activity.

Cellular RNA helicases have been reported to be involved in HCV genome replication. DDX3 and DDX6 activate HCV genome replication through yet unknown mechanism [29], [30]. RNA helicase p68 (DDX5) interacts with NS5B and supports HCV genome replication in a transient transfection assay [31]. Although the mechanism through which each RNA helicase regulates HCV genome replication may be different, the requirement of cellular RNA helicases for HCV genome replication is interesting for understanding HCV-cellular factors interaction.

CAHL expression in normal liver cells was much less than that in HCV infectious cells such as Huh-7 and MH-14. This is enigmatic since it is not clear how HCV replication start without CAHL, which positively plays HCV replication, at very beginning of HCV infection in normal liver cells. It was reported that a proinflammatory cytokine, TNF-α gene expression in hepatocytes and mononuclear cells derived from HCV carrier increased compared with healthy control [32]. As we here demonstrated CAHL induced by TNF-α, CAHL can express to some extent in the liver under chronic hepatitis C. We also show the association of CAHL with HCV replication. Taken together, CAHL may form a positive feedback loop for HCV replication: CAHL gene expression is induced by TNF-α that is highly upregulated by HCV infection, and CAHL in turn promotes HCV replication. Despite the low expression of CAHL in normal tissues, CAHL may have strong potential as a pharmaceutical target protein. In addition to CsA, isolation of specific inhibitors to the interaction of CAHL and NS5B could allow us to provide effective drug for HCV treatment.

In conclusion, we took advantage of strategy of chemical biology to isolate a cellular factor, CAHL, as CsA associated helicase-like protein, which would form trimer complex with CyPB and NS5B of HCV. These findings not only shed a light on new HCV treatment but also brought about great values of chemical biology to elucidate biological mechanisms of small-molecule and protein interactions.

## Materials and Methods

### Preparation of CsA-immobilized resins

CsA was purchased from Wako Pure Chemical Industries, Ltd. (Osaka, Japan). CsA-immobilized resins were prepared on photoaffinity resins as described previously [12]. Photoaffinity resins treated with UV irradiation in the absence of CsA were used for negative control.

### Phage display screening

10 mg of CsA-immobilized resin was incubated in 1 ml of TBS (50 µM Tris-HCl [pH 8.0] and 150 mM NaCl) for 12 hours or longer before use. Phage screening conditions were performed as previously described [20]. For each panning step, 50 µl of CsA-immobilized resin slurry was added to 1 ml of the T7 phage (>10^11^ pfu) followed by incubation for 8 hours at 4°C. After incubation, the bead slurry was washed 10 times by adding 1 ml of TBST (50 mM Tris-HCl [pH = 8.0], 150 mM NaCl, 0.1% Tween 20). To elute phage particles associated with resins, 100 µl of *Escherichia coli* (OD_600_ = 0.6) was added, and incubated for 10 min at 37°C. The phage infected *E. coli* were transferred into 1 ml of *E. coli* (OD_600_ = 0.6) and grown until lysed for three hours at 37°C with shaking. For titer check, 10 µl of infected *E. coli* was used.

### RNA preparation and plasmid construction

To isolate the CAHL gene, we used total RNA derived from human liver. DNA cloning of the CAHL gene was carried out using a SMART-cDNA isolation kit following the manufacturer's instructions (Clontech Laboratories, CA, USA). In some cases, CAHL cDNA was reconstructed with pcDNA 3.1 myc-HisA (Invitrogen Corp., CA, USA) for overexpression experiments.

### Surface plasmon resonance assay

SPR analysis was performed on a BIAcore 3000 (Biacore AB, Uppsala, Sweden). The bacterially expressed CAHL-C was immobilized covalently on a hydrophilic carboxymethylated dextran matrix on a CM5 sensor chip (Biacore AB) using a standard amine coupling reaction in 10 mM CH_3_COONa [pH = 4.0]. Binding analyses were carried out in HBS-EP buffer (10 mM HEPES [pH = 7.4], 150 mM NaCl, 3.4 mM EDTA, 0.005% surfactant P20) containing 8% DMSO at a flow rate of 20 µl/min. Appropriate concentrations of CsA were injected over the flow cell. CyPA or CyPB was not used as a positive control because of two reasons: 1) those CyPs can be used for a positive control as CsA-CyP binding, but not for CsA-CAHL binding, and 2) FK506 doesn't bind CyPs, so that it is difficult to compare association behaviors between FK506 and CsA. The bulk effects of DMSO were subtracted using reference surfaces. To derive binding constants, data were analyzed by means of global fitting using BIAevaluation version 3.1 (Biacore AB).

### Preparation of recombinant protein

CAHL-C cDNA encoding C-terminal 761 to1430 amino acids was constructed into pET21a prokaryotic expression vector (Merck, Darmstadt, Germany), which has a His-tag. pET21- CAHL-C construct was transformed into *E.coli* BL21(DE3) strain. After overnight induction with 0.1 mM IPTG at 20°C, recombinant CAHL-C was purified by nickel column chromatography with HisTrap (Amersham biosciences, Uppsala, Sweden) according to a manufacturer's procedure. To concentrate and exchange the buffer, purified CAHL-C was concentrated up to 40 times with PBS by using Amicon Ultra 30 (Millipore, EMD, Germany).

### ATPase assay

ATPase activity was measured as described by Okanami *et al*. [33]. Briefly, CAHL-C protein (500 ng) was incubated in 50 µl of helicase/ATPase buffer containing 1 µl of [γ-^32^P]ATP (1 Ci/µmol) in the presence or absence of 100 ng of total RNA derived from liver at 30°C for 30, 60, and 180 min. An aliquot (10 µl) was removed at the appropriate time and added to 200 ml of a solution containing 50 mM HCl, 5 mM H_3_PO_4_ and 7% activated charcoal. After the charcoal was precipitated by centrifugation to remove unreacted ATP, 10 µl of the supernatant was subjected to Cerencov counting to quantitate released [^32^P]phosphate.

### Northern blot analysis and reverse transcription PCR (RT-PCR) analysis

Tumor cell-derived total RNA was prepared using an RNeasy Mini Kit (QIAGEN Inc., Hilden, Germany) according to the manufacturer's instructions and then reverse-transcribed to cDNA with Transcriptor First Strand cDNA Synthesis Kit (Roche Applied Science, Mnnheim, Germany). A reverse-transcribed single strand DNA library of normal tissues was purchased from Clontec, Inc. (CA, USA). In Northern blot analysis, RNA samples were loaded to formaldehyde agarose gels and transferred onto a Hybond N membrane (GE Healthcare UK Ltd., Buckinghamshire, England). After UV-crosslinking, the membrane was hybridized with ^32^P-labeled (Rediprime II, GE Healthcare) gene-specific probe, regions of CAHL_2913-4431_ and human G6PDH_454-2016_ and exposed to film for autoradiography. Measurement of CAHL gene expression by polymerase chain reaction (PCR) was performed using GoTaq Flexi DNA Polymerase (Promega, Co. WI, U.S.A.) and primer sets: forward primer, 5′-GACGGGAAAGGATTGGTCAA-3′ and reverse primer, 5′-CATCACTTCGTGCTTTTT-3′ for detection of CAHL, and forward primer, 5′-GACGAAGCGCAGACAGCGTCATGGCA-3′ and reverse primer, 5′-GCTTGTGGGGGTTCACCCACTTG-3′ for detection of G6PDH.

### Quantitative real-time RT-PCR analysis

Total RNAs reverse-transcribed to cDNA were prepared as described above. Measurement of gene expression by quantitative analysis was performed using the LightCycler system (Roche Applied Science). Primers and hybridization probes were synthesized by Nihon Gene Research Laboratory Inc. (Sendai, Japan). Quantitative real time RT-PCR analyses of human glucose-6-phosphate dehydrogenase (G6PDH) and cyclosporin A associated helicase-like protein (CAHL, NM_022828) gene expression were performed using the LightCycler® FastStart DNA MasterPLUS SYBR Green I system (Roche Applied Science) with the following primer sets: forward primer, 5′-CTGCGTTATCCTCACCTTC-3′ and reverse primer, 5′-CGGACGTCATCTGAGTTG-3′ for detection of human G6PDH; forward primer, 5′-GTGTCTGGACCCCATCCTTA-3′and reverse primer, 5′-CCCATCACTTCGTGCTTTTT-3′for detection of CAHL. Gene expression analysis of the HCV genome was performed using the LightCycler® FastStart DNA Master HybProbe system (Roche Applied Science) with the following primer set and probe: forward primer, 5′-CGGGAGAGCCATAGTGG-3′ and reverse primer, 5′-AGTACCACAAGGCCTTTCG-3′, and the fluorogenic probe, 5′-CTGCGGAACCGGTGAGTACAC-3′. PCR amplification of the housekeeping gene, G6PDH, was performed for each sample as control for sample loading and to allow normalization among samples. To determine the absolute copy number of the target transcripts, the fragments of G6PDH or target genes amplified by PCR using the above described primer set were constructed with pCR4®-TOPO® cloning vector (Invitrogen). The concentrations of these purified plasmids were measured by absorbance at 260 nm and copy numbers were calculated from concentration of samples. A standard curve was created by plotting the threshold cycle (Ct) versus the known copy number for each plasmid template in the dilutions. The copy numbers for all unknown samples were determined according to the standard curve using LightCycler version 3.5.3 (Roche Applied Science). To correct for differences in both RNA quality and quantity between samples, each target gene was first normalized by dividing the copy number of the target by the copy number of G6PDH, so that the mRNA copy number of the target was the copy number per the copy number of G6PDH. The initial value was also corrected for the amount of G6PDH indicated as 100% to evaluate the sequential alteration of the mRNA expression level.

### Cell culture and transfection of siRNA and cDNA

The human tumor cell lines of breast adenocarcinoma MDA-MB-231, lung adenocarcinoma A-549, colon adenocarcinoma WiDr, hepatocellular carcinoma Huh-7, breast cancer SKBR3, cervical carcinoma HeLa, esophagus cancer KE-4, colon adenocarcinoma SW480, lung cancer Lu65, and esophagus squamous cell carcinoma TE-8 were obtained from Health Science Research Resources Bank (Sendai, Japan). These cells were cultured in Dulbecco's modified Eagle's medium (Huh-7, SKBR3, HeLa, KE-4, and SW480 cells), RPMI 1640 (A-549, WiDr, TE-8, and Lu65 cells) (SIGMA-ALDRICH, MO, USA), and Leibovitz's L15 (MDA-MB-231 cells) (Invitrogen) supplemented with 10% fetal bovine serum, MEM nonessential amino acids (Invitrogen), 200 unit/ml penicillin (Invitrogen), 200 µg/ml streptomycin (Invitrogen) and 2 mM L-glutamine (Invitrogen). MH-14 cells carrying the HCV subgenomic replicon [34] were cultured in the DMEM medium supplemented with 10% fetal bovine serum, MEM nonessential amino acids (Invitrogen), 200 unit/ml penicillin (Invitrogen), 200 µg/ml streptomycin (Invitrogen), 2 mM L-glutamine (Invitrogen) and 300 µg/ml G418 (Invitrogen). Five small interfering RNA (siRNA) duplexes containing 3′dTdT over the hanging sequence were synthesized (Sigma-Aldrich, St. Louis, MO). These sequences were: si-1; 5′-GGACAUUCGCAUUGAUGAG-3′, si-2; 5′-CCUGUAAUUUGACUCAUAA-3′, si-3; 5′- GCCUUGGAUGUAAAUCUCUUU -3′, si-4; 5′- GGAGCUUUCAGUGACCAUA -3′, si-5; 5′-GGUCAAAUAAUAGUAGGAA-3′. A non-targeting siRNA (Sigma-Aldrich) was used as control. Plasmid and siRNA transfection was performed described previously [35]. In siRNA study, total RNAs from transfected cells were harvested after transfection for 5 days and examined mRNA copy number of CAHL by quantitative real-time RT-PCR.

### Establishment of stable CAHL-knockdown cell by shRNA

Based on the siRNA data, we applied short hairpin RNA (shRNA) technology platform (Sigma Mission*RNAi*) to stably knockdown CAHL gene expression in MH14 cells. DNA oligo coding the effective siRNAs against each MH14-CAHL gene (5′- CCGGGCCTTGGATGTAAATCTCTTTCTCGAGAAAGAGATTTACATCCAAGGCTTTTTTG -3′) (sh-CAHL) was cloned into pLKO.1-puro shRNA vector. Plasmid DNA including non-targeting shRNA as control (sh-control) was transfected into MH14 cells along with Lentiviral Packaging Mix consisting of an envelope and packaging vector (Sigma-Aldrich) to produce lentivirus packed with shRNA cassettes using the standard procedure. After transfection, cells were cultured in the presence of 10 µg/ml puromycin.

### Indirect immunofluorescence analysis

Anti-CAHL polyclonal antibody serum was generated in rabbits immunized with CAHL_1237-1251_, ILHPKRGTEDRSDQS, according to our lab protocol [35]. Anti-NS3, NS4B, and NS5B, and NS5A were kindly provided from Dr. Kohara at The Tokyo Metropolitan Institute of Medical Science, Japan and Dr. Takamizawa at Osaka University, Japan, respectively. Cells were fixed with ice-cold acetone for 1 min, and then stained with anti-CAHL and anti-KDEL mAb (Santa Cruz Biotechnology, CA, USA) for ER antibodies followed by Alexa Fluor 488-conjugated goat anti-rabbit IgG and 594-conjugated goat anti-mouse IgG (Invitrogen), respectively, and visualized using a Bio-Rad MRC1024ES laser confocal scanning microscopy system (Bio-Rad Laboratories, CA, USA).

### Immunoblot analysis

Immunoblot analysis was performed essentially as described previously [11], [36].

### RNA-protein binding precipitation assay

RNA-protein binding precipitation assay was essentially performed as described [11]. Briefly, to permeabilize plasma membrane, cells were treated 50 µg/ml digitonin (Nakarai Tesque Inc., Kyoto, Japan) in buffer B (20 mM HEPES-KOH [pH = 7.7], 110 mM KOAc, 2 mM MgOAc, 1 mM EGTA) at 25°C for 5 min. After treatment with 0.5 µg/ml proteinase K at 37°C for 5 min and washing with buffer B, cells were lysed in IP buffer (50 mM Tris-HCl [pH = 8], 150 mM NaCl, 0.5% NP-40, and protease inhibitory cocktail [Roche Applied Science]). After centrifugation, supernatants were incubated for 2 h with poly-U or protein G Sepharose resin (GE Healthcare). After four washes with IP buffer, precipitates were analyzed by immunoblot analysis. Supernatants after centrifugation were used as a positive control (designated Cell lysate in Fig. 4E and 4D).

### GST-pull-down assay

GST-pull-down assay was performed as described previously [36].

## Supporting Information

Figure S1 Predicted amino acid sequences of CAHL (NM_022828). Underlined residues (LLGQLRA) indicate identical sequence of phage clone #13.
(TIFF)

Figure S2 Indirect immunoflourescence analysis for colocalized with between CAHL and NS3, NS4A, NS4B, NS5A, and NS5B using Huh-7 (A) and MH-14 (B). The primary antibodies used were anti-CAHL (panels a, d, g, j, and m, green) and anti-NS proteins (panels b, e, h, k, and n, red) antibodies. Marge images of green and red signals are shown in panels c, f, i, l, and o.
(TIFF)

Figure S3 Determinant of knockdown efficiency against CAHL gene expression. Five siRNAs for the CAHL gene were individually transfected into MH-14 cells. After transfection, total RNAs of these cells were collected and examined mRNA copy number of CAHL by quantitative real-time RT-PCR.
(TIFF)

Table S1 List of phage clones and their encoding deduced peptide sequences screened by CsA biopanning. *Asterisk indicates the identical sequences.
(DOCX)
