# Supplementary figures and images for: Cyclosporin A Associated Helicase-Like Protein Facilitates the Association of Hepatitis C Virus RNA Polymerase with Its Cellular Cyclophilin B
Source: PLoS One. 2011 Apr 29;6(4):e18285. doi: 10.1371/journal.pone.0018285 (PMC3084704; doi:10.1371/journal.pone.0018285)

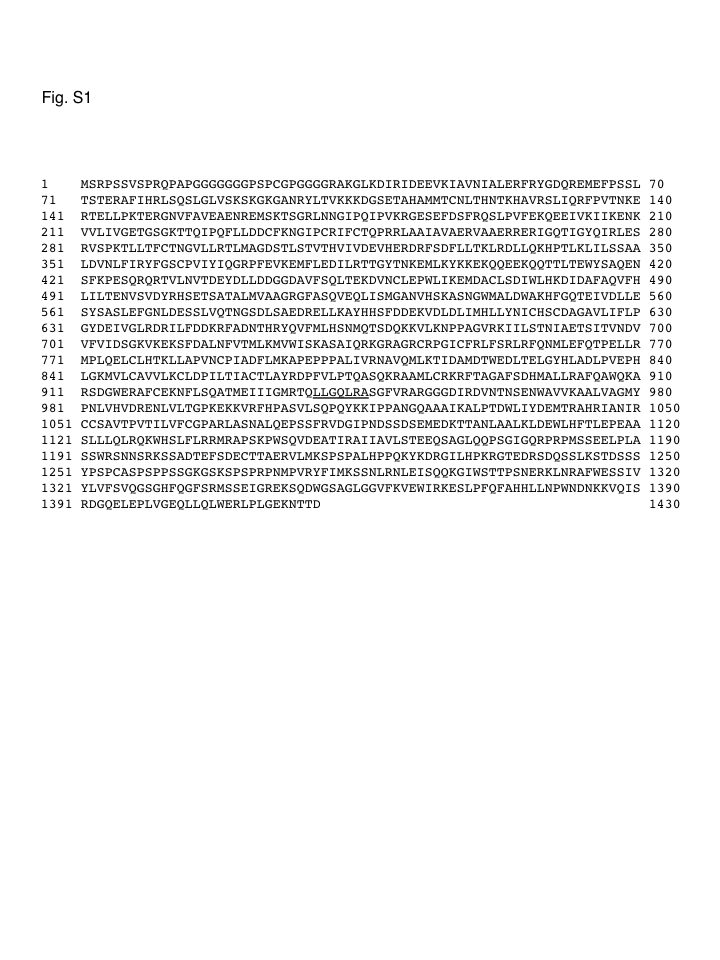

Supplement: Figure S1 — Predicted amino acid sequences of CAHL (NM_022828). Underlined residues (LLGQLRA) indicate identical sequence of phage clone #13. (TIFF) [file pone.0018285.s001.tif]

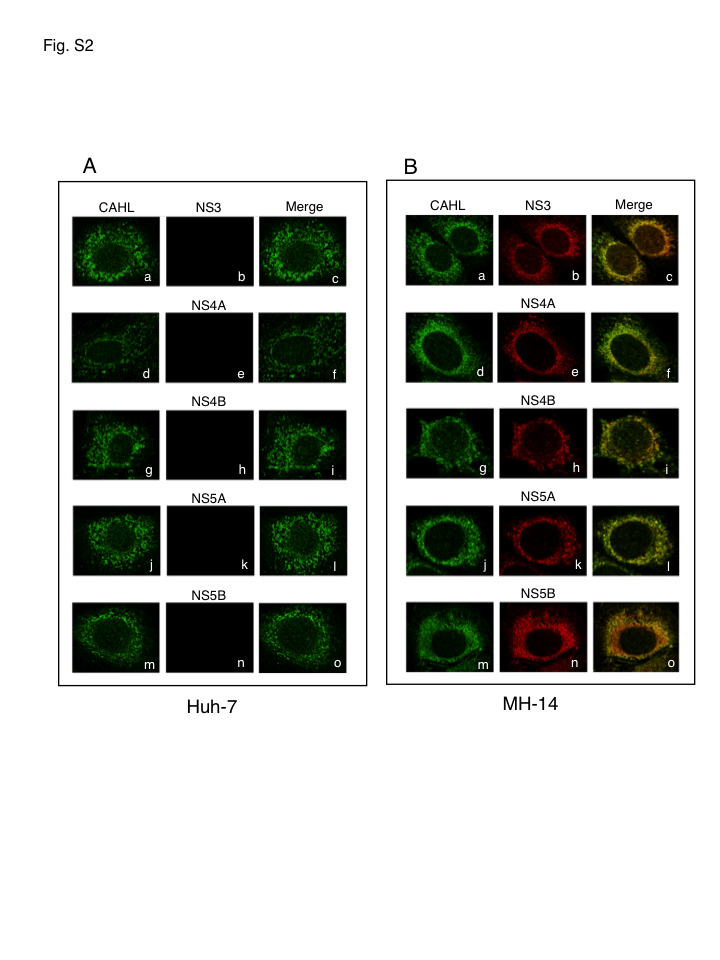

Supplement: Figure S2 — Indirect immunoflourescence analysis for colocalized with between CAHL and NS3, NS4A, NS4B, NS5A, and NS5B using Huh-7 (A) and MH-14 (B). The primary antibodies used were anti-CAHL (panels a, d, g, j, and m, green) and anti-NS proteins (panels b, e, h, k, and n, red) antibodies. Marge images of green and red signals are shown in panels c, f, i, l, and o. (TIFF) [file pone.0018285.s002.tif]

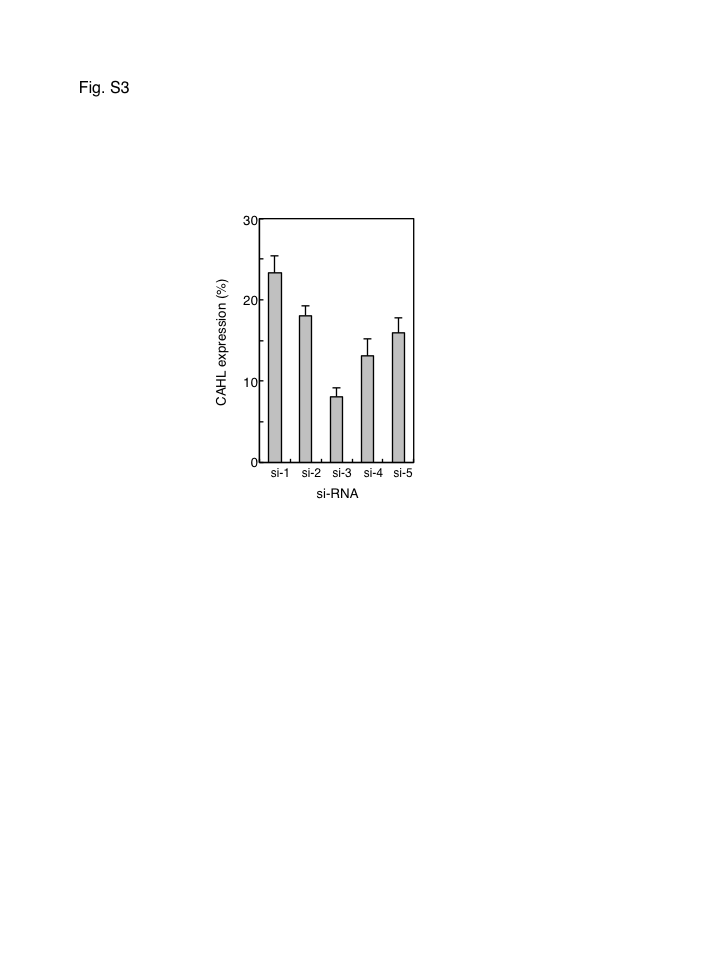

Supplement: Figure S3 — Determinant of knockdown efficiency against CAHL gene expression. Five siRNAs for the CAHL gene were individually transfected into MH-14 cells. After transfection, total RNAs of these cells were collected and examined mRNA copy number of CAHL by quantitative real-time RT-PCR. (TIFF) [file pone.0018285.s003.tif]
