# Supplementary material for: Cyclosporin A Associated Helicase-Like Protein Facilitates the Association of Hepatitis C Virus RNA Polymerase with Its Cellular Cyclophilin B
Source: PLoS One. 2011 Apr 29;6(4):e18285. doi: 10.1371/journal.pone.0018285 (PMC3084704; doi:10.1371/journal.pone.0018285)
Supplement: Table S1 — List of phage clones and their encoding deduced peptide sequences screened by CsA biopanning. *Asterisk indicates the identical sequences. (DOCX) [file pone.0018285.s004.docx]

Table S1. List of phage clones and their

encoding deduced peptide sequences

screened by CsA biopanning.

| Clone number | Amino acid sequence |
| --- | --- |
| 1 | F |
| 2 | PPSYLVLTGDSS |
| 4 | LSDHATFWASKV |
| 5 | SVQYV |
| 7 | FICIFRSSSVCG |
| 11 | LPV |
| 13 | LVFGTLLGQLRA* |
| 14 | CL |
| 15 | VSPSYYSWWNFR |
| 16 | FAQMVIATNLSEM |
| 18 | NVSFR |
| 20 | LVFGTLLGQLRA* |
| 21 | LVFGTLLGQLRA* |
| 22 | NVSFR |
| 23 | LVFGTLLGQLRA* |
| 24 | F |
| 26 | CFMRL |
| 27 | VSPSYYSWWNFR |
| 28 | LVFGTLLGQLRA* |
| 32 | VSPSYYSWWNFR |
| 33 | IFVVLHFVCVHA |
| 36 | F |

*Asterisk indicates the identical sequences.
